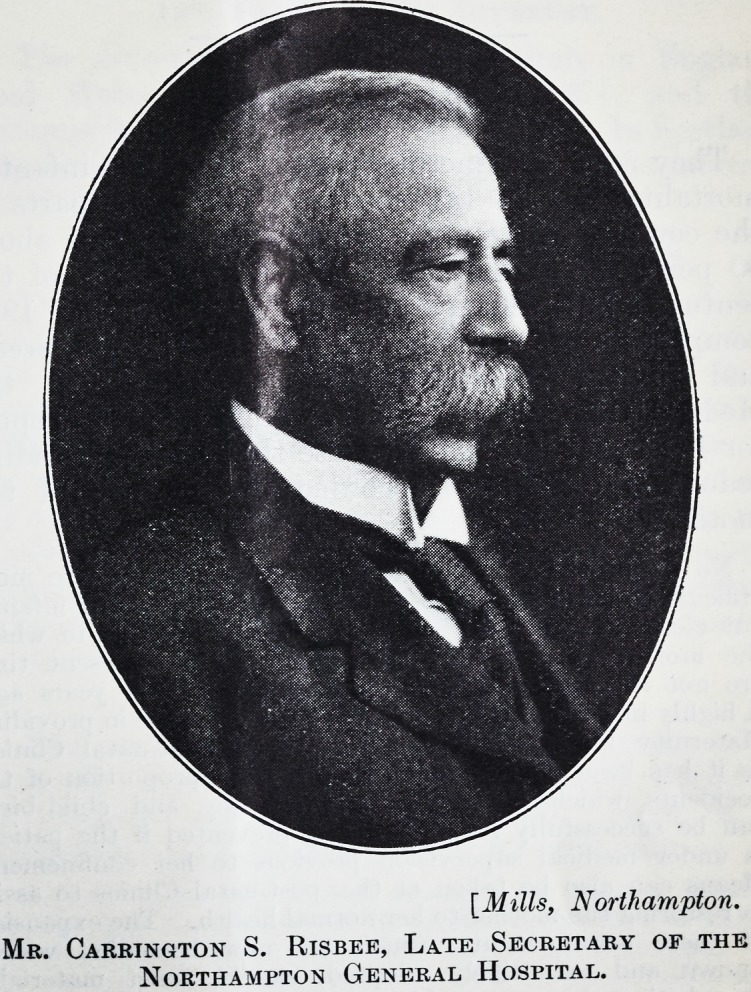# Hospital Men of Mark: Mr. Reginald B. Loder and Mr. Carrington S. Risbee

**Published:** 1924-11

**Authors:** 


### HOSPITAL MEN OF MARK.

### MR. REGINALD B. LODER AND MR. CARRINGTON S. RISBEE.

The Northampton General Infirmary was founded in 1743, and from very small beginnings has risen to be one of the best equipped provincial hospitals of the present day. Its position is particularly desirable, since on the south it is free from buildings and has the immense advantage of being entirely open to the country, while on the north it is immediately accessible to the town. Since 1897, the date of Mr. Risbee's appointment as secretary, an appointment which, owing to advancing years, he has recently felt compelled to resign, many changes have taken place, and the annual income has increased from £7,800 to £35,000. In 1901 a committee was appointed to consider the condition of the wards and the inadequate accommodation in the old Infirmary. Sir Henry Burdett was consulted on the question and plans were adopted for the erection of two new wings to contain 156 beds, making the old Infirmary into a nurses' home and an administration block. Towards the scheme Sir Henry Burdett and one of the architects offered a joint donation of £1,000 on the condition that promises of nine other donations of £1,000 each were obtained. Within five weeks this sum and £700 over were forthcoming.

*Illingworth, Northampton.]*

Mr. R. B. Loder, President of Northampton General Hospital.

*[Mills, Northampton.*

Mr. Carrington S. Risbee, Late Secretary of the Northampton General Hospital.

The name of the Infirmary was changed in 1903 to the Northampton General Hospital, and in 1904 the new wings were opened by Earl Spencer, and Mr. Risbee was appointed to the post of superintendent as well as that of secretary. After inspecting the buildings Sir Henry Burdett congratulated the Board and the Governors upon the possession of "one of the best, most modern and up-to-date of county hospitals, which will always possess the merit of having been completed at a cost within the original estimate. The care, forethought and supervision which have been exercised by the Building Committee and others deserve the generous acknowledgments of the town and county of Northampton."

Since that date the work of the hospital has gone steadily forward. More beds have been added, a pathological laboratory and an ophthalmic out-patient department equipped, and a convalescent home opened, while new isolation wards are being built. In 1921 a central heating station was installed at a cost of £18,000, replacing fifteen old type boilers that were distributed all round the building. In every department of its activities the president and chairman, Mr. Reginald B. Loder, has keenly interested himself. A prominent resident in the county and a member of the Pytchley Hunt Committee, he has for twenty-four years been chairman of the Finance Committee. He is not only himself a generous supporter, but through his influence with members of the Hunt has from time to time obtained from them large donations and annual subscriptions. He has for many years taken the greatest personal interest in the management and welfare of the institution, and its eminent position and sound financial standing are mainly due to Mr. Loder's keen supervision and exceptional knowledge of finance.

In the retirement of Mr. Risbee Northampton sustains a heavy loss. He carries with him the good wishes of all his fellow-workers and the knowledge that he leaves the hospital in a high state of efficiency. "When he came here," said Mr. Loder recently, "he found a moderate-sized infirmary, and he leaves a large county hospital. He found a poorly-equipped establishment; he leaves what I have no hesitation in saying is a well-equipped one. He found one Standing Committee; at the present time there are no fewer than sixteen. When he came here he was single-handed, now he has five clerks to assist him, and they are fully occupied and can scarcely cope with the work." Mr. Risbee was for twelve years a member of the British Hospitals Association. He was succeeded upon his resignation of that position by Mr. Loder. Mr. Risbee's successor, as we have already announced, is Mr. H. St. John Wood, secretary of the West Kent Hospital, Maidstone.

## Figures and Tables

**Figure f1:**
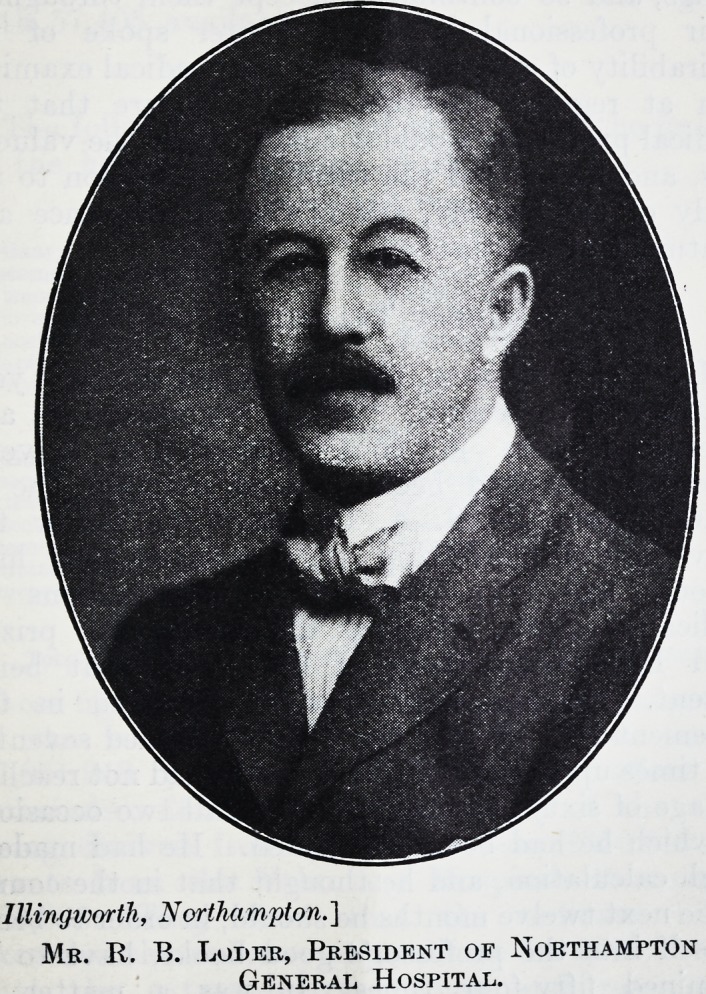


**Figure f2:**